# Effect of Interpenetrating Polymer Network (IPN) Thermoplastic Resin on Flexural Strength of Fibre-Reinforced Composite and the Penetration of Bonding Resin into Semi-IPN FRC Post

**DOI:** 10.3390/polym13183200

**Published:** 2021-09-21

**Authors:** Minori Hatta, Akikazu Shinya, Harunori Gomi, Pekka K. Vallittu, Eija Säilynoja, Lippo V. J. Lassila

**Affiliations:** 1 Department of Crown and Bridge, School of Life Dentistry at Tokyo, The Nippon Dental University, 1-9-20, Fujimi, Chiyoda-ku, Tokyo 102-8159, Japan; m-hatta@tky.ndu.ac.jp (M.H.); h.gomi@tky.ndu.ac.jp (H.G.); 2 Department of Dental Materials Science, School of Life Dentistry at Tokyo, The Nippon Dental University, 1-9-20, Fujimi, Chiyoda-ku, Tokyo 102-8159, Japan; 3 Department of Prosthetic Dentistry and Biomaterials Science, Institute of Dentistry, University of Turku, Lemminkaisenkatu 2, 20520 Turku, Finland; pekval@utu.fi (P.K.V.); eija.sailynoja@gc.dental (E.S.); liplas@utu.fi (L.V.J.L.); 4 Research Development and Production Department, Stick Tech Ltd.—Member of GC Group, 20520 Turku, Finland

**Keywords:** fibre-reinforced composite, interpenetrating polymer network, fibre post, post and core, flexural strength, PMMA

## Abstract

The purpose of this study was to evaluate the effects of interpenetrating polymer network (IPN) thermoplastic resin on the flexural strength of fibre-reinforced composite (FRC) with different IPN polymer compositions. The penetration of bonding resin into semi-IPN FRC posts was also evaluated. The IPN thermoplastic resin used was UDMA-MMA monomer with either PMMA (0.5%, 2%, 5%) or PMMA-copolymer (0.5%, 2%). A no added IPN polymer resin was also made. Mixed resin was impregnated to S- and E-glass fibre rovings. These resins and resin impregnated fibres were used for flexural strength (FS) test. To evaluate the penetration of bonding resin into semi-IPN post, SEM observation was done with various impregnation time and polymerization mehods (hand-light- and oven-cure). The result of FS was recorded from 111.7 MPa (no-IPN polymer/no-fibre-reinforcement) to 543.0 MPa (5% PMMA/S-glass FRC). ANOVA showed that there were significant differences between fibre-reinforcement and no-fibre-reinforcement (*p* < 0.01) both in S- and E-glass fibre groups, and between 0.5% PMMA and 5% PMMA in the S-glass FRC group. SEM micrographs showed that the penetration layers of bonding resin into hand-light cured semi-IPN posts were different according to impregnation time. Fibre reinforcement is effective to improve flexural strength. The depth of penetration layer of bonding resin into semi-IPN matrix resin was improved when a hand-light cure was used.

## 1. Introduction

Fibre-reinforced composite (FRC) is a composite material which consists of reinforcing fibres embedded in a resin polymer matrix. FRCs have been developed for decades and are widely applied in dental treatment [1]. FRCs are commonly used for direct and indirect restorations, such as fixed partial dentures (FPDs) [2,3], root-canal posts [4], periodontal splinting [5] and orthodontic applications [6]. Thus, FRCs have now become good alternatives to conventional metal-based treatments and also resolve not only patients’ esthetic problems, but cost and time considerations.

The resin matrix used in FRCs is typically composed of a highly cross-linked polymer such as bis-phenol A diglycidyl ether dimethacrylate (Bis-GMA), triethylene glycol dimethacrylate (TEGDMA), or urethane-dimethacrylate (UDMA) [7]. In addition, another concept, that of interpenetrating polymer networks (IPNs), has been introduced. This is a polymer comprised of two or more kinds of polymer in network form, with continuous mutual interlacing [8]. This IPN polymer has been also applied to, for example, FRC root canal posts and FRC frameworks of FPD [9]. Semi-IPN for dental polymer matrices is composed of polymethylmethacrylate (PMMA) and bis-GMA, and also achieves a chemical bonding between the FRC materials and luting cements or veneering composite [10].

Many types of fibres are used clinically, however, the most commonly used ones are E-glass fibres and S-glass fibres which can be silanized, and adhere well to the resin matrices of FRCs [9]. Another reason is their translucency which makes it easy to adjust the tooth color and/or outer materials, and satisfies the patient’s esthetic demands [11].

For mechanical testing, three-point bending tests have been universally used to test the flexural properties of composite materials. Previously, many studies which compare cross-linked polymer FRC to IPN FRC, have been done and the mechanical properties of FRC materials evaluated [4,12,13]. The fracturing of FRC caused by biting forces should be avoided, especially in the posterior molar areas. It is well known that when occlusal forces are loaded onto FRC structures, stresses are transferred from the matrix to the reinforcing fibres to function. Therefore, we need to carefully consider not only the fibres but the stiffness of the matrix resin itself.

For FRCs, the impregnation of the matrix resin into every fibre becomes one of the most important factors to prevent FRCs from fracturing or failing. If complete impregnation and polymerization are not achieved, void spaces appear and then the occlusal forces cannot be transferred to fibres, and failure may occur [14,15]. Thus, reinforcing fibres and resin matrices should be tightly combined together. However, the commercial FRC materials which form IPNs are quite limited, and the effects of the compositions of IPN polymer in the resin matrix have not been evaluated much. Moreover, it is still not clear how the different compositions of IPN polymers added to resin matrices affect the mechanical strength of FRCs.

The good bonding of resin materials to the polymer matrix resin is also a very important part in the fabrication of FRC FPD or adhesion of luting cement to a dentin surface. In previous studies, the penetration depths of bonding resin into resin matrices were reported [16], and it was proved that the penetration progressed into deeper areas of the matrix resin of semi-IPN FRC than in a cross-linked polymer FRC [10,17]. Moreover, the penetration layers after various impregnation periods were evaluated and their effects have been clarified [18]. However, commercial semi-IPN post (everStick post in this study) needs to be polymerized using light curing or oven curing units, therefore, the differences of penetration layers polymerized by these two methods are worth evaluating. This may also be useful information for selecting the fabrication method for oven-cured laboratory-made restorations.

The present study was thus conducted to evaluate the effects of IPN thermoplastic resin on the flexural properties of FRC with various IPN polymer compositions of PMMA or PMMA-copolymer to UDMA-MMA-based resin matrices. In addition, the penetration of bonding resin into resin-impregnated semi-IPN FRC post with two different polymerization methods (hand-light cure and oven cure) was also evaluated using scanning electron microscopy (SEM).

## 2. Materials and Methods

In this study, fibre-reinforced composite (FRC) with various combinations of IPN thermoplastic resin were prepared with two different kinds of fibres (S- and E-glass). All materials used in this study are listed in Table 1. The thermoplastic resin was based on urethane dimethacrylate (UDMA, Esschem, Linwood, PA, USA)-methyl methacrylate (MMA, Sigma-Aldrich, St. Louis, MO, USA) monomer system. For the interpenetrating polymer network (IPN)-forming polymer either polymethyl methacrylate (PMMA, Mw 150 kD, Sigma-Aldrich, St. Louis, MO, USA) or PMMA-copolymer (Mw 150 kD, Sigma-Aldrich, St. Louis, MO, USA) was used. The experimentally used polymer matrix resins were prepared according to the IPN polymer content (PMMA: 0.5%, 2%, 5%, PMMA-copolymer: 0.5%, 2%). A no-IPN polymer added resin was also prepared as control. Thus, six different contents of IPN thermoplastic resin were available for flexural strength (FS, three-point bending) testing. The compositions of all the kinds of resin are shown in Table 2.

In addition, to observe the penetration condition of resin primer into FRC, everStick posts (StickTech-GC, Turku, Finland) which are adaptable, polymer (PMMA) and resin-impregnated (bis-GMA) unpolymerized glass fibre posts, composite primer (GC, Tokyo, Japan, CP), and diiodomethane for labeling the primer were selected and subjected to scanning electron microscopy (SEM) observation. These materials are also listed in Table 1.

### 2.1. Fabrication of Specimens for the Flexural Strength Tests

#### 2.1.1. Preparation of IPN Thermoplastic Resins

Six different polymer contents of experimentally used matrix resin mixture were prepared. First, 10 g of MMA was measured using an automatic scale (ME 403, Mettler Toledo, Columbus, OH, USA), and put it into the empty storage glass bottle, and a total of six MMA (10 g) bottles were prepared. Then, IPN polymer of either PMMA or PMMA-copolymer was added to each bottle, respectively, according to the polymer content (0.5 g, 2 g, 5 g of PMMA and 0.5 g, 2 g of PMMA-copolymer). The bottles with MMA-IPN polymer were placed on a mixing device and mixed homogeneously until it was verified that no visible polymer was in the bottle. Thereafter, 90 g of UDMA was added to the mixed monomer/polymer resin in the bottles and mixed again continuously. After UDMA was completely mixed in, photocuring initiator/activator (0.7 g of camphorquinone, CQ, Sigma-Aldrich, St. Louis, MO, USA) and 2-(dimethylamino) ethyl methacrylate (DMAEMA, Sigma-Aldrich, St. Louis, MO, USA) were added and mixed again for one night in the dark. Also, no-IPN polymer (0%) added UDMA-MMA-DMAEMA-CQ resin was also prepared for control. Thus, total six different compositions of resin mixture were ready for subsequent specimen preparation. The same weighing scale was used throughout this study.

#### 2.1.2. Fabrication of Specimens for Flexural Strength Tests

**Figure 1:**
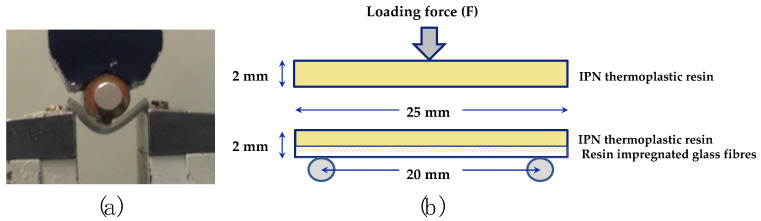
3-point bending (FS) test (**a**) and the schematic drawings of specimens from front side (**b**): upper; No fibre reinforced specimen and lower; fibre reinforced specimen: a bundle of S2 or E resin impregnated glass fibres is placed on the bottom (tensile) side. Specimen size is 2 mm × 2 mm × 25 mm. The distance between two supports is 20 mm.

Using the mixed IIPN resin, rectangular bar-shaped flexural strength (FS, three-point bending) test specimens of 2 × 2 × 25 mm size were fabricated (Figure 1). In total 180 specimens were fabricated for the FS tests. They were divided into three main groups: (1) specimens with no fibre-reinforcement (n = 10 × 6 subgroups), (2) specimens with S2-glass fibre-reinforcement (n = 10 × 6 subgroups), and (3) specimens with E-glass fibre-reinforcement (n = 10 × 6 subgroups).

For no fibre-reinforcement specimens (Group1), a stainless-steel mold with a 2 × 2 × 25 mm cavity was placed on a glass plate and a plastic translucent film, then the mixed resin was directly put in the cavity of the mold using a plastic syringe so that the no air bubbles formed in the resin. The surface of the resin-filled mold was covered with another translucent plastic film and glass plate. Then, the resin was hand-light cured for 60 s using a LED hand curing device (Elipar S10, wavelength: 430–480 nm, power: 1200 mW/cm^2^ intensity, 3M, St Paul, MN, USA). After hand-light curing, the polymerized specimen was removed from the mold. All hand light-cured specimens were then oven cured (TargisPower, IVOCLAR, Schaan, Liechtenstein) for 25 min at 96 °C for final polymerization. The light curing time was set to 60 s in this study. According to the manufacturer of the commercial everStick IPN FRC materials, at least 40 s of light curing is required for direct or indirect use of these materials. In this study, length of specimens is longer than clinical use of everstick products, so that light curing time was set for this study. The additional post-curing time and temperature were determined from a Targis/Vectris combination which was one of the FRC fixed partial dentures. This final polymerization condition was based on previous studies by Abdulmajeed et al. [19] and Nganga et al. [20].

For fibre-reinforcement specimens, we used the same resins shown in Table 2 for impregnation of fibre rovings and FRC specimen fabrication. A bundle of S2- and E-glass fibres (tex 2400, MCX21, Owens-Corning, Toledo, OH, USA) was cut to a length of 25 mm, and the fibre rovings was impregnated with each resin between the plastic films for 30 min in the dark. After this process, the resin-impregnated fibre rovings were placed on the bottom of the cavity of the mold. The empty space of the cavity was filled with the same resin for impregnation. The polymerization of this fibre-resin composite was carried out same method as no-fibre reinforcement specimen preparation.

All hand-light and oven cured (no fibre-reinforcement and fibre-reinforcement) specimens were left to evaporate for 24 h. All fabricated specimens were then polished by #500, #800 and #1200 silicon carbide grinding paper with an automatic polishing machine under water to remove excess resin and to obtain the appropriate size. The polished specimens were dried spontaneously and stored at room temperature for 24 h under dry conditions before mechanical testing.

#### 2.1.3. Flexural Strength (FS, Three-Point Bending) Tests

Before starting the FS tests, the width and thickness of the loading point of the specimens were measured again using a digital caliper and the size entered into the test program. The measured specimen was fixed to the mounting jig, and the load was applied in the air using an universal testing machine (Lloyd LRX, Lloyd Instruments Ltd., Bognor Regis, UK) with a crosshead speed of 1.0 mm/min and loading span of 20 mm until fracture. FS was calculated using the following formula:

FS = 3Fl/2bh^2^

where F: applied load (N) at maximum point of the load-deflection curve, l: span length of the specimen, b: width of the specimen and h: thickness of the specimen.

#### 2.1.4. Statistical Analysis

All results were statistically analyzed by two-way analysis of variance (ANOVA) and Tukey’s multiple comparison test at the significant level of 0.05. This study was carried out about two factors. One is according to the different compositions of matrix resin and the other one is according to with and without fibre reinforcement.

### 2.2. Fabrication Methods of Specimens for SEM Observation

The FRC posts used for SEM analysis specimens were everStick posts (0.9 mm diameter, StickTech GC, Turku, Finland). First, CP (1.0 g) and diiodomethane (Sigma-Aldrich, St. Louis, MO, USA, 0.1 g, 10 wt% of CP) were measured and mixed. This mixture was divided into two plastic cases. Then, two bundles of everStick 0.9 mm posts were combined together by hand and eight similar posts were fabricated so that the diameter of each fibre post was 1.8 mm. Four fabricated 1.8 mm posts were hand-light cured for 60 s and the remaining four posts were oven-cured for 25 min at 96 °C before immersion. Each polymerized post was immersed into the mixed primer and stored for 1 min, 5 min, 1 h, and 24 h, respectively. After the appropriate immersion time has passed, the post was removed from the primer. Gradia Core (GC) was mixed with a Gradia Core dispenser gun equipped with a GC Automix Tip (Gradia, GC, Tokyo, Japan), and filled it into the plastic ring (5 mm diameter). Each post was inserted into the center of the cement-filled ring mold. These embedded post and resin were hand-light cured for a total of 80 s from four directions. The light-cured post-resin specimen was removed from the ring mold. Excess post was cut off with a diamond disc and hand polished using #800 silicon carbide paper to clean resin off the post cross-section. These fabricated specimens were subjected to SEM observation with a magnification of 250×. The penetration depth was evaluated from the iodine-colored areas.

## 3. Results

### 3.1. Flexural Strength

**Figure 2:**
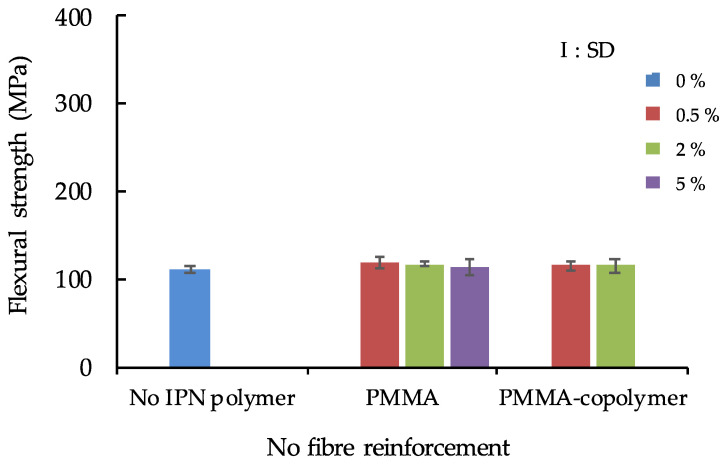
Flexural strength of IPN thermoplastic resin with no fibre reinforcement.

**Figure 3:**
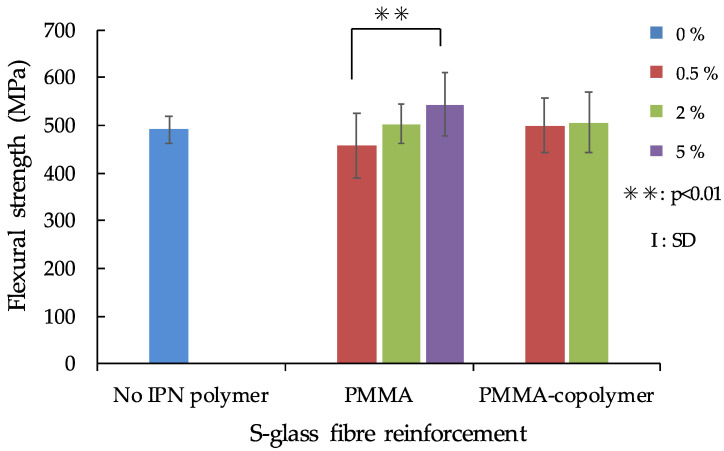
Flexural strength of IPN thermoplastic resin with S-glass fibre reinforcement.

**Figure 4:**
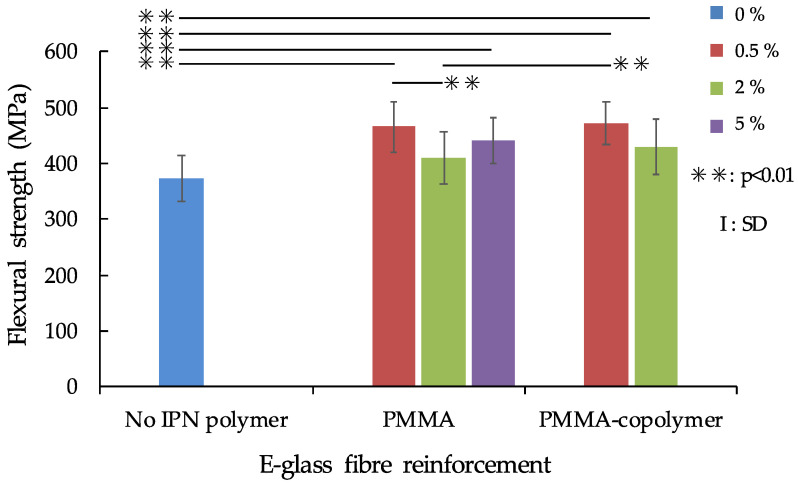
Mean flexural strength of IPN thermoplastic resin with E-glass fibre reinforcement.

The flexural strength results of the IPN thermoplastic resin with no fibre reinforcement are shown in Figure 2, while the flexural strength of IPN thermoplastic resin with S-glass fibre reinforcement is shown in Figure 3. The maximum flexural strength value of FS was recorded in 5% PMMA/S-glass FRC (543.0 MPa), and the minimum value (111.7 MPa) was observed in no-IPN polymer/no-fibre reinforcement samples. ANOVA showed that there were significant differences between fibre reinforcement and no fibre-reinforcement (*p* < 0.01) in both the S- and E-glass fibre groups. For the S-glass fibre reinforcement group, there was a significant difference only between 0.5% PMMA and 5% PMMA (Figure 3), however, the difference of IPN polymer (PMMA and PMMA-copolymer) did not influence the FS. On the other hand, for the E-glass fibre reinforcement group, IPN polymer contents of PMMA and PMMA-copolymer influenced the FS values as shown in Figure 4. 

### 3.2. SEM Observation

**Figure 5:**
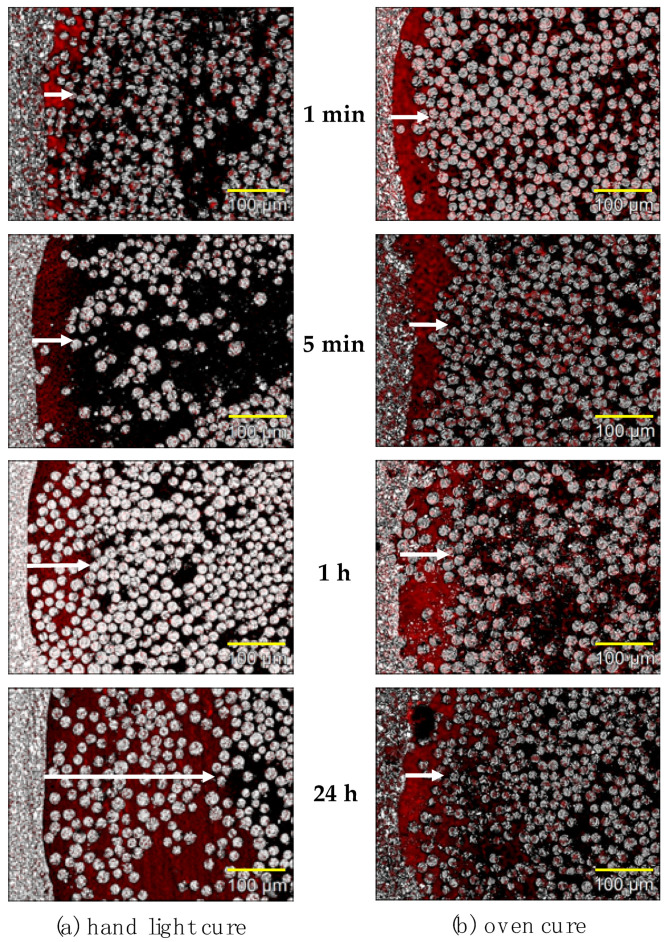
SEM photographs of the penetration conditions of priming agents into everStick POST: (**a**) hand light cured after 1 min, 5 min, 1 h, and 24 h, (**b**) oven cured after 1 min, 5 min, 1 h, and 24 h of immersion time.

SEM photographs of the penetration of CP into polymerized everStick posts revealed with diiodomethane are shown in Figure 5. As shown in Figure 5a, hand-light cured specimen shows almost no penetration layer after 1 min immersion, however, after 5 min and 1 h one layer of penetration is seen. After 24 h, the penetration layer of CP was observed in a deeper layer and everywhere in the specimen. On the other hand, the oven-cured specimens did not show a noticeable penetration layer after different immersion times ranging from 1 min to 24 h as shown in Figure 5b.

## 4. Discussion

This experimental study demonstrated the effects of various interpenetrating polymer network (IPN) matrices on the flexural properties of fibre-reinforced composite (FRC) made using S-and E-glass fibres. Whether the different compositions of additional IPN polymer (PMMA or PMMA-co-polymer) changed the flexural strength was evaluated. The differences in bonding resin penetration layers in hand-light and oven-cured semi-IPN FRC resin matrices were also evaluated by SEM.

In the present study, several compositions of experimentally laboratory-mixed urethane-dimethacrylate (UDMA, 90%)-methyl-methacrylate (MMA, 10%) monomer-based resin were prepared. Presently, several kinds of monomers are applied in dental practice, for example, Bis-GMA, TEGDMA and UDMA [21,22]. Of these, an UDMA-MMA based monomer system was used. An IPN forming polymer of PMMA or PMMA-copolymer (poly(styrene-co-methyl methacrylate)) to dimethacrylate resin were also added. PMMA has been used in dentistry for many years as a denture base material. The thermoplastic PMMA denture base polymer has been made by mixed MMA monomer liquid and PMMA beads [9]. When PMMA-powder and MMA-monomer liquid are polymerized, semi-IPN is formed [23]. Furthermore, this IPN polymer of PMMA has been also applied to FRC, and some studies were conducted not only by using commercial products like the everStick system but experimentally prepared IPN-FRCs [13,24]. Another PMMA-copolymer was also used as IPN forming polymer in this study. This was also applied to denture base material, and addition of styrene caused an increase in the values of the flexural strength and hardness [25]. Based on these experimental reports, PMMA and PMMA-copolymer were chosen.

In the preparation of matrix resin mixture, IPN polymer of either PMMA or PMMA-co-polymer and MMA were mixed first. Then this resin dough was further mixed with UDMA. During this process, a technical difficulty occurred during PMMA-copolymer/UDMA-MMA dough mixing. PMMA-copolymer compositions higher than 5% were completely dissolved in MMA, so 5% PMMA-copolymer-MMA dough was not homogeneously mixed with the UDMA. It might be considered that the polymerization of PMMA-copolymer-MMA had started before it was fully mixed in the UDMA. Therefore, the matrix resin of 5% PMMA-co-polymer of-UDMA-MMA group was rejected. PMMA is usually used as a denture base resin [26], and when the volume of PMMA powder is increased, the viscosity of the PMMA-MMA mixture also increases, as one of the roles of PMMA is to adjust the viscosity of the resin dough [22]. At the same time, PMMA-co-polymer is a copolymer of PMMA and polystyrene, so, the inclusion of polystyrene might affect something during resin mixing. Considering this mixing process, we set up the IPN polymer content to 0.5%, 2%, and 5% of PMMA, 0.5% and 2% of PMMA-copolymer. The cause of this was not clarified this study. Further consideration of the mixing method used for laboratory-made resins will be needed.

FRC specimens fabricated using the laboratory-made resin mixture were subjected to a 3 point bending test for the flexural strength tests. The fibre materials used were S- and E-glass fibres. Several types of fibres are applied in dental practice [1,11], and one of the reasons to choose S- and E-glass fibres in this study was that they have advantages of translucency, in addition to excellent mechanical properties [4,11]. Also, E-glass fibre is commonly used in FRC materials with an IPN resin matrix. The original fibre roving was pre-impregnated with each resin mixture. To our knowledge, one of the success points of FRC materials when used in an oral environment is the good adhesion between each fibre and the resin matrix. If this resin impregnation into the fibres is not sufficient, stresses applied to resin matrix are not transferred to the reinforcing fibres and as a result, fractures or defects may occur in the final products. In this study, a bundle of fibre roving was impregnated with each resin carefully using a syringe prior to final polymerization. Less information about the impregnation method was found for laboratory-made resins. Some studies have been discussed impregnation times for intact fibres, however, the applied pre-impregnation times varied from one hour to 24 h [13,27]. In this study, used 30 m as the impregnated fibre roving became translucent. Further consideration about the appropriate impregnation time will be needed to improve any clinical application.

The flexural strength (FS) results of the no-fibre reinforcement group were shown in Figure 2. In this group, no significant differences were recorded between all the compositions including no (0%) IPN polymer and IPN polymer-added groups. The FS of the no- IPN polymer added group showed the lowest value. Therefore, for the no-fibre reinforcement composite which has traditional resin monomer system and IPN composite resin, it was clear that additional IPN polymer did not affect the flexural strength in this study. Moreover, the FS of all fibre-reinforced groups showed significantly higher values than those of the no-fibre reinforcement groups (*p* < 0.01). Therefore, first of all, it was clear that using S- or E-glass fibres is effective for the reinforcement of matrix resins in this study. Three point bending tests were used in this study. When evaluating the materials’ bonding properties, we often use shear bond strength tests. However, the 3-point bending test is also applied as a testing method. This is typically used in experiments related to the assessment of porcelain-fused to metal crowns [28,29,30], where the bond strength between porcelain and metal is assessed by using 3 or 4 point bending tests. Therefore, we followed this concept.

As shown in Figure 3, in FS of S-glass FRCs, there was a significant difference only between the 0.5% PMMA and 5% PMMA added groups. In both the PMMA and PMMA-copolymer added groups, the FS tended to increase as the IPN polymer composition increased. On the other hand, the E-glass fibre group has shown different phases. As shown in Figure 4, the FS of no-IPN polymer recorded the lowest value, and there were significant differences between IPN polymer-added groups except for the 2% PMMA group. This means that addition of IPN polymer influenced the FS values. Comparing to the no fibre-reinforcement group results shown in Figure 2, the FS of fibre-reinforcement with IPN polymer matrices showed various changes. With the addition of polymers to resin matrices and further combination with fibres, FRC-IPN might have an effective use for making improved IPN thermoplastic resin materials. For FRC framework materials, a previous study showed that FS of semi-IPN FRC recorded a higher value (796 MPa) than a cross-linked polymer FRC (689 MPa) [12]. Another study also reported that semi-IPN FRC tended to give a higher FS (1150 MPa) than a cross-linked UDMA-based matrix FRC (1005 MPa) in dry conditions, and also even after thermal cycling [31]. Furthermore, in FRC root canal post experiments, a study has shown that a semi-IPN FRC post recorded a higher FS than that of a commercial cross-linked polymer FRC post [4]. However, it is not always possible to directly compare the FS values with previous studies, because the mechanical properties are dependent on the resin matrix components and the fibres used, fibre orientation, the ratio of fibre to resin matrix, the adhesion between the fibres and resin matrix, and so on [11]. Further studies will be needed to evaluate the effects of the composition of IPN polymers.

To analyze the penetration of bonding resin into the IPN matrix resin, semi-IPN (everStick) posts were used. To visualize the penetration layer, 10 wt% of diiodomethane was mixed with CP. In some studies, confocal laser scanning microscopy was used for detecting the penetration layer with rhodamine-B-isothiocyanate as fluorescent dye [10,17]. In this study, we used SEM, and the penetration layer could be clearly observed with colored diiodomethane. This would be useful and effective option to evaluate not only the penetration like in this study but also marginal gaps in restorations and dentin/enamel interfaces instead of commonly used X-rays.

The penetration depth of bonding resin into the polymer matrix of semi-IPN FRC and cross-linking polymer (CLP) FRC posts has already been reported. The bonding resin more effectively penetrated deeper into a semi-IPN resin matrix than a CLP matrix [10,17].

In this study, we set up various bonding resin impregnation times: 1 m, 5 m, 1 h and 24 h. For use of bonding resin in chair-side direct treatment, 1 m and 5 m of bonding time are acceptable both for patients and operators, whereas longer impregnation times like 24 h will not be practical. On the other hand, a previous report showed that a longer impregnation time of 24 h led to better penetration into deeper areas of the resin matrix [18]. This result led to higher bond strength in veneering composites [18]. That is, the overlaying materials are firmly interlocked with semi-IPN FRCs. Although a longer impregnation time of 24 h can only be applied to laboratory-made products, we also set up various longer impregnation time up to 24 h as the possible maximum impregnation time.

In the SEM micrographs of the hand-light cure (Figure 5a), the penetration of bonding resin into semi-IPN FRC matrix resin has been seen in a deeper layer with the progress of impregnation time. After 1 min of impregnation only the surface layer was penetrated by bonding resin as compared to other longer times. Mannocci et al. [10] showed that the penetration of bonding resin could be noted after 30 s of impregnation time, and was always noted after 300 s. This supports our present study results. It might be considered that 1 min impregnation in this study is enough for clinical use, especially in chair-side direct treatments, however, for safety, 300 s and more should be recommended. On the other hand, a maximum 24 h of impregnation showed that the penetration of bonding resin into the deeper inner layer of semi-IPN resin matrix was still proceeding. This 24 h period can be applied to laboratory-made restorations, if higher bonding properties to a veneering composite and/or luting cement are necessary because a thicker penetration layer results in certain interlocked IPN bonding. On the contrary, when using the oven- cure method (Figure 5b), the penetration did not progress during the longer impregnation time. SEM micrographs, however, showed that the layer after 1 m was almost the same as after 1 m of hand-light cure. These results may be related to the degree of monomer conversion in the matrix resin. Alander et al. showed that the degree of monomer conversion in a resin matrix resulted lower when using hand-light curing rather than oven-curing [32]. Good bonding between FRC and resin monomers or veneering resins is generally related to the existence of an unpolymerized surface layer, the so-called oxygen inhibition layer [17]. Also, the CP used in this study contains hydroxyethyl methacrylate (HEMA). The surface of the matrix resin of hand-light cured specimens might have more space for bonding resin penetration. On the other hand, for oven cure specimens, the surface was cured by heating and the post might become similar to a hard cross-linking post. In previous studies it was shown that this HEMA-containing bonding resin could effectively dissolve the linear phases of semi-IPN matrices [16,17] and then, the bonding properties were increased [16,18]. In this study, bond strength was not evaluated, however, it will be needed to clarify the effects of bonding resin, HEMA, after using these two polymerization methods.

## 5. Conclusions

- In the S-glass fibre-reinforcement group, there was a tendency to increase the flexural strength according to the increase of IPN polymer composition, however, no statistical differences were shown except between 0.5% and 5% PMMA.
- In the E-glass fibre-reinforcement group, additional IPN polymers significantly influenced the increase in flexural strength, except for 2% PMMA.
- The depth of the penetration layer of bonding resin into the semi-IPN matrix resin was improved when hand-light curing was used, and oven-cured posts did not show any improvement with impregnation time.

## Figures and Tables

**Table 1:** Materials used in this study.

Material	Components	Manufacturer
IPN thermoplastic resin		
UDMA	Urethane-dimethacrylate	Esschem
MMA	Methyl-methacrylate	Sigma-Aldrich
CQ	Camphorquinone	Sigma-Aldrich
DMAEMA	2-(Dimethylamino) ethyl methacrylate	Sigma-Aldrich
PMMA	Poly (methyl methacrylate)	Sigma-Aldrich
PMMA-copolymer	Poly (Styrene-co-methyl methacrylate)	Sigma-Aldrich
Glass fibres		
S2-glass fibre		Owens Corning
E-glass fibre		Owens Corning
SEM observation		
everStick Post 0.9 mm	PMMA, BisGMA (Bis-phenol A diglycidyl ether dimethacrylate), E-glass fibres	StichTech-GC
GRADIA CORE	UDMA	GC
COMPOSITE PRIMER	UDMA, HEMA(2-hydroxyethyl metacrylate)	GC
Diiodomethane		Sigma-Aldrich

**Table 2:** Composition of IPN thermoplastic resin.

Code	UDMA	MMA	PMMA	PMMA-Copolymer	CQ	DMAEMA
0% IPN polymer	90%	10%	−	−	0.7%	0.7%
0.5% PMMA	90%	10%	0.5%	−	0.7%	0.7%
2% PMMA	90%	10%	2%	−	0.7%	0.7%
5% PMMA	90%	10%	5%	−	0.7%	0.7%
0.5% PMMA-copolymer	90%	10%	−	0.5%	0.7%	0.7%
2% PMMA-copolymer	90%	10%	−	2%	0.7%	0.7%

## Data Availability

Data of the study are available on request form the corresponding author.
